# Designing for Multilevel Behavior Change: A Father-Focused Nutrition and Physical Activity Program for Mexican-Heritage Families in South Texas Border Communities

**DOI:** 10.3390/ijerph181910117

**Published:** 2021-09-26

**Authors:** Cassandra M. Johnson, Joseph R. Sharkey, M. Renée Umstattd Meyer, Luis Gómez, Marlyn A. Allicock, Tyler Prochnow, Elva Beltrán, Luz Martinez

**Affiliations:** 1 Nutrition and Foods Program, School of Family and Consumer Sciences, Texas State University, San Marcos, TX 78666, USA; 2 Department of Health Promotion and Community Health Sciences, School of Public Health, Texas A&M University, College Station, TX 77843, USA; jrsharkey@tamu.edu (J.R.S.); gomez@tamu.edu (L.G.); beltran@tamu.edu (E.B.); lmartinez@tamu.edu (L.M.); 3 Department of Public Health, Robbins College of Health and Human Sciences, Baylor University, Waco, TX 76706, USA; renee_umstattd@baylor.edu; 4 The University of Texas Health Science Center, Department of Health Promotion and Behavioral Sciences, Houston School of Public Health-Dallas Regional Campus, Dallas, TX 75207, USA; marlyn.a.allicock@uth.tmc.edu; 5 Department of Health and Kinesiology, College of Education and Human Development, Texas A&M University, College Station, TX 77843, USA; tprochnow@tamu.edu

**Keywords:** colonias, promotoras, Latino fathers, family systems, health promotion, rural

## Abstract

Fathers significantly influence family functioning, as coparents and partners, and must be part of family-based approaches to behavioral health interventions or programs. But little is known regarding how to support Latino fathers in health promotion within their family systems, specifically for Latino families living in border communities. Program development was embedded in a larger community-based grant and part of a longstanding academic-community collaboration. An interdisciplinary research team applied theories related to health behavior, family systems, behavior change, and community engagement to develop a father-focused and family-centered behavioral program for Mexican-heritage fathers and children living near the Texas-Mexico border to support changes in nutrition and physical activity at the individual and family levels. Promotoras de salud (trained community health workers) delivered the program through group sessions, check-in calls, and at-home activities. Group session activities were designed to engage family triads and dyads using experiential education related to nutrition and physical activity, like cooking lessons and active play, over a six-week period. Future research can use the program approach and curricula as a roadmap for designing context-specific and culturally-relevant programs for Latino families. Additional research is needed to explore how approaches like this can support families and their health goals.

## 1. Introduction

Prior research suggests focusing on parents as the agents of change in childhood obesity prevention and treatment may be more effective than child-focused interventions or programs [1]. Several programs have targeted improved diet and activity behaviors in children through parental modeling or other parent-child interactions [2,3,4]. Researchers have suggested going beyond parenting styles or behaviors to target more broad family influences, such as family functioning or dynamics. The idea is that programs can achieve more meaningful impacts by focusing on family stress, cohesion, and parenting challenges [5,6]. At the same time, family-based programs can no longer ignore fathers. Nearly all family-based programs, including those targeting parenting behaviors around food and activity, have focused on mothers [7,8,9,10]. Fathers significantly influence family functioning, as coparents and partners, and must be part of family-based approaches to programs. Toward that goal, there has been a shift to family-centered approaches, which engage the entire family [11,12]. In contrast, family-based or family-friendly programs engage individual family members, such as a parent or a child, or perhaps a couple of family members, such as a family dyad like a mother-child pair. In contrast, a family-centered approach values fathers as coparents and partners and considers the family as a system to address broader family functioning and affect more meaningful and sustainable changes within family systems [11,12]. A family systems approach may promise improved effectiveness with low-income or ethnic-minority families [11].

There is growing interest and need for family-centered and father-focused behavioral health programs to support nutrition and physical activity within family systems [9,12,13,14,15,16]. Particularly, with Latino families living in border communities and colonias [17], there is a critical need to support health-promoting behaviors related to nutrition and physical activity. Communities along the Texas-Mexico border are occupied by a growing population of people who share a similar Mexican heritage, language, and socioeconomic standing; have unacceptably high rates of persistent poverty and financial stress, adult and childhood obesity, food insecurity, and physical inactivity; and limited access to affordable, healthy foods and physical activity opportunities [18,19,20,21]. Recently, authors have published an article describing strategies to support Latino fathers in obesity prevention programs, with specific strategies for every stage of the process, that is, initial recruitment, retention, and engagement, and the active and maintenance phases of behavioral programs [22]. But, to our knowledge, there has been no family-centered and father-focused program developed for and with Latino families, especially for families living in border communities. In addition, leaders at the National Institute of Health and Minority Research (NIMHD) have highlighted family dynamics and family norms as important behavioral determinants for health outcomes at the family level in their research framework [23]. They have also emphasized a need for “novel approaches” that are “multilevel and multifactor” to address social, cultural, and environmental influences that drive health disparities [24]. Further, scholars have described the unrealized potential of multi-level programs for advancing the science of health disparities and promoting health equity [25]. The current paper describes the design of the ¡*Haz Espacio para Papi*! (HEPP, Make Room for Daddy!) program for Mexican-heritage families in border communities in South Texas, which aimed to positively change nutrition and physical activity behaviors among participating fathers and their children. The purpose of this manuscript is to describe the rationale and design, including the theoretical foundation, and offer a roadmap for future behavioral programs with this priority population.

## 2. Materials and Methods

### 2.1. Context

An interdisciplinary and multi-institution team developed the HEPP program as part of the overall *Salud para Usted y Su Familia* (SPUSF, Health for You and Your Family) initiative that integrated research, education, and outreach to improve family and community health. The team included academic-based researchers in public health with training and experience in nutrition, physical activity, social and behavioral health, a licensed family psychologist with expertise in Latino family systems, and promotoras (specially trained community health workers who worked as research partners [26]), with training and experience in education, social work, and research. Individual team members represented different genders, racial and ethnic backgrounds, and prior experience with community-based research and practice. 

Community-engaged research (CEnR) principles guided the development process [27,28]. The program was created simultaneously in English and Spanish with an iterative process of development and review. Program development was part of a long-standing collaboration with stakeholders and communities in South Texas, and specific engagement with three community advisory boards in Progreso, San Juan, and San Carlos and prior outreach, research, and education activities with residents and the team of promotoras [29,30]. Previously published articles have described the collaboration with “promotora-researchers” in outreach, research, and education [26,29,30]. 

In addition, program development was based on extensive formative work. The research team conducted formative research in the community to understand needs, assets, desires, and preferences for a behavioral program focused on nutrition and physical activity. In preparation for this program, the field-based research team identified, mapped, and ground-truthed 16 geographic areas or “clusters” to describe the neighborhood environments and community resources. Clusters were defined based on similarity in size, population, sociodemographics, and naturally occurring boundaries. A sequence of activities with Mexican-heritage families followed ground-truthing: Children’s Pláticas (3-session panel series of discussions of 12 groups of children, and one one-on-one interview as a make-up session, related to nutrition, physical activity, and family relationships), Mothers’ Pláticas (3-session panel series of discussions with seven groups of mothers, and one one-on-one interview make-up session), Mothers’ Elicitation surveys (*n* = 334), Fathers’ Dyadic Interviews (facilitated discussion between two fathers; *n* = 31), and Fathers’ Elicitation surveys (*n* = 330). The children’s and mothers’ pláticas integrated participant-driven photo-elicitation [29]. All participants provided consent to be contacted for additional studies. 

The formative work revealed the importance of fathers’ engagement and their encouragement in food and physical activity choices. Mothers spoke of their desire for greater father engagement and the father being a role model but admitted they were reluctant to create opportunities for father involvement due to not knowing where to start. Fathers, on the other hand, shared they were eager to provide their insights since they, as fathers, felt were rarely given the opportunity to participate in similar programs or be heard. Nonetheless, they were ready to learn how to be more involved in ways that were meaningful to their own families and serve as role models. The dyadic interviews provided fathers with an opportunity to reflect on their roles as husbands and fathers. They acknowledged the value of coparenting as a couple-team with their partners or spouses. Fathers described a willingness to step out of their gender roles to please their children and the importance of family. A second manuscript, which we also plan to submit for review to this special issue, details the multi-phase approach to engaging promotoras de salud (promotoras, specially trained community health workers) through the design, implementation, and evaluation of HEPP. 

### 2.2. Approach

The HEPP program was a father-focused, family-centered, strengths-based program designed to initiate and support behavior change at the individual and family levels. While the program targeted nutrition and physical activity behaviors, the program primarily focused on nutrition. The literature guided the team’s approach to father engagement [7,12,31,32,33,34], design and implementation of family-centered programs (health promotion and disease prevention/treatment) [9,11,12], including programs with Latino families, specifically those led by Ayala and colleagues [35,36,37], and our prior research with the community [26,29,30], including formative work related to this grant (see section called Context). The approach also integrated a promotora model, as a team of promotoras (community health workers) collaborated on program design, implementation, and evaluation [35,36]. 

### 2.3. Theoretical Foundation

**Figure 1:**
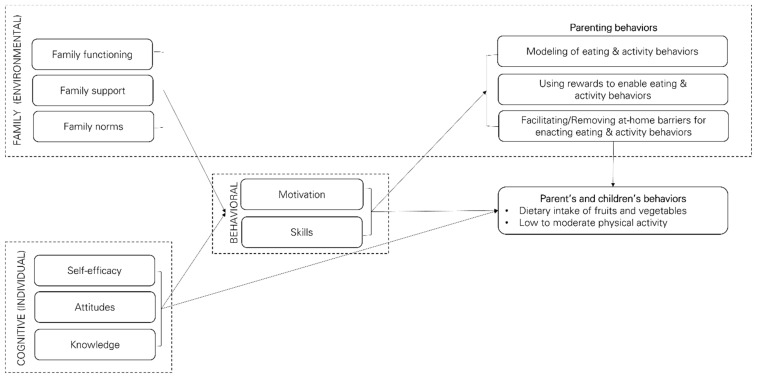
Conceptual model for multilevel behavioral program the ¡Haz Espacio para Papi! (HEPP, Make Room for Daddy!) A detailed description of the theoretical foundation for this conceptual model is provided in the section called Theoretical Foundation. This conceptual model includes multi-level factors at the individual, behavioral, and environmental levels.

The HEPP program drew on several theories, such as Social Cognitive Theory (SCT) [38], Family Ecological Model (FEM) [39], the Family Systems Theory (FST) [40], the Family-Centered Action Model of Program Layout and Implementation (FAMILI) model [11], and the Circumplex Model of Marital and Family Systems [41] to support multi-level behavior change at the individual and family levels with the priority population. Theories were selected for appropriateness for a program targeting individuals within family systems and environments, and based on the literature (e.g., previous father- or family-focused behavioral programs). Prior programs, such as Ayala et al. *Entre Familia: Reflejos de Salud*, that used a “whole family approach” also focused on targeting family functioning and parenting behaviors related to food [35,37]. Specifically, the HEPP program aimed to change dietary intake of fruits and vegetables and physical activity at the individual and family level within Mexican-heritage families. The theories provided a framework that underscored the importance of family interactions within the home or household environment. Figure 1 presents the conceptual framework for HEPP. 

As described in the SCT, reciprocal determinism exists between individual, behavioral and environmental factors and underscores the need to consider how individuals influence and are influenced by their environments [38]. As outlined in the FEM: “parents influence children’s physical activity and dietary behaviors as a result of their knowledge and beliefs related to diet, physical activity, and body weight. In turn, parents’ knowledge and beliefs influence the extent to which they model healthy and unhealthy behaviors, use feedback to shape children’s diet and activity behaviors, and facilitate children’s access to environments that promote healthy or unhealthy eating and physical activity” [11] (p. 457). The Family-centered Action Model of Program Layout and Implementation or FAMILI blends two theories: the Family Systems Theory (FST) and the Family Ecological Model (FEM) [11]. 

According to the conceptual framework, program activities at the individual level would change individual cognitive and behavioral factors, which would directly change nutrition-related behaviors of children and parents (dietary intake of fruits and vegetables and low to moderate physical activity). The primary individual determinants are knowledge, attitudes, self-efficacy, personal motivation, and skills related to enacting nutrition behaviors, and the individual behaviors themselves: dietary intake of fruits and vegetables and low to moderate physical activity. At the same time, program activities at the family level would change overall family functioning, family support, and family norms, which would change parenting behaviors related to nutrition and physical activity, especially modeling, and indirectly affect children’s behaviors. The program used the Circumplex Model to define family functioning as cohesion, flexibility, and communication [41]. Applying the FAMILI approach [11], HEPP incorporated opportunities to develop: (1) “constancy feedback loops” in place of negative feedback loops to maintain existing health-promoting traditions, and (2) “variety feedback loops” to add new ideas and behaviors. The importance of feedback loops, guided by FAMILI, informed the program’s focus on embracing existing health-promoting traditions and encouraging new healthy traditions. 

At the individual level, guided by SCT, the HEPP aimed to change cognitive factors like individual self-efficacy, attitudes, and knowledge. From SCT, self-regulatory strategies like goal setting, self-monitoring, providing feedback, and problem-solving are used to achieve behavior change in children, and parents, both fathers, and mothers. At the family level, the HEPP aims to change environmental factors related to family functioning, which is attributed to FEM. This program targeted family functioning, defined by cohesion, flexibility, and communication between family members [41], and focused on parent-child relationships. Prior research and family-focused childhood obesity programs guided the focus on family-level health determinants [3,35,42,43,44]. Table S1 provides selected examples of how the program operationalized and targeted some of the theoretical constructs. 

The program included three program activities: in-person group sessions, check-ins (phone calls and home visits), and at-home activities between group sessions. The research team hypothesized that the experiential education and skills-building during the in-person group sessions would change individual and behavioral factors, including motivation and skills related to dietary intake and physical activity. Problem-solving and goal-setting activities were hypothesized to change motivation and skills and support behavior change. Self-monitoring of dietary and physical activity behaviors, completed as part of the at-home activities, were hypothesized to affect change of family-level norms and support. The check-ins provided additional opportunities for problem-solving and goal-setting customized to each family. Co-participation in nutrition and physical activity, where parents and children jointly engaged in an activity, was a vital part of HEPP. According to the conceptual framework, family activities related to nutrition and physical activity behaviors, completed during in-person group sessions and at-home activities, would affect change in overall family functioning, family norms and support, and parent-child interactions related to parenting, which are sometimes described as “food parenting” [45]. Key family activities planned for the in-person group sessions included: strengthening family dynamics through the interactive lessons, cooking together, engaging in active play during physical activity breaks, eating together, and goal setting. Throughout the program, there were opportunities for positive reinforcement modeled through group leaders’ interpersonal interactions and incentivized with raffles for participants who engaged in at-home activities individually and as a family. Cues to action were incorporated in the program through tangible materials, such as the Family Guide, and small items to promote family cooking and eating together as well as exercise and active play (Table S1). At the end of the program, families took home the complete cooking toolkit, which included cutting boards, mixing bowls, chef’s knives for adults and children, and other kitchen tools to support at-home food preparation.

### 2.4. Recruitment and Retention

The program intentionally recruited and engaged Mexican-heritage fathers in addition to mothers. Panter-Brick and colleagues have suggested that engaging another parent-in-the-home, like mothers, supports retention [12]. In collaboration with promotoras and community partners, potential participants were identified through various methods commonly used in community-engaged research. For example, promotoras recruited families from those who had previously participated in the formative work, flyers and word of mouth, and going door to door in the study area. During recruitment, promotoras verbally stressed the uniqueness of the program and the program’s value to fathers and the entire family. Additionally, promotoras highlighted the flexibility of the program sessions and how sessions would be adapted to work around their work schedules and other common barriers (e.g., literacy levels, interest in topics and activities, inclusive learning environment, etc.) identified by Mexican-heritage fathers. Prior literature on family-centered and father-focused programs has emphasized the importance of designing programs that overcome barriers to participation from the fathers’ perspectives and communicating the value of fathers’ participation to fathers and mothers, including the costs and benefits of participation [7,8,9,12,16,34].

The program aimed to recruit 10 to 12 families from each cluster or geographically-defined neighborhoods in the study area. Eligible families were identified using the following inclusion criteria: parents 21 years old or older and self-identifying as Mexican-heritage (participant, parent, or grandparent born in Mexico), cohabitating with partner or spouse and a child between 9 and 11 years at enrollment (or the start of the program), able to complete in-home measurement visits pre-and post-program, able to commit to full participation in the 6-week program, parents preferred to speak, read, and write in Spanish, and parents had to have lived in the colonia (or neighborhood cluster) for at least one year. Exclusion criteria included: parents or participating child reporting a severe food allergy or reporting physical activity restrictions [32,42,46]. 

The literature also has discussed the importance of providing transportation and childcare to support program attendance [32,34]. The HEPP program provided childcare, but only provided transportation as needed. Promotoras helped coordinate transportation for families on a case-by-case basis. Providing on-site childcare was essential. Paid staff and volunteers supervised non-participating children and offered games, arts and crafts activities, and recreational activities during all group sessions. Activities were developed and available for all ages, from infants to teenagers.

### 2.5. Informed Consent Process

The research team developed an informed consent process in collaboration with the promotoras and based on literature [47]. For example, the team created informed consent forms (in Spanish and English-language) at or below a fifth-grade reading level and incorporated visual aids and graphics to improve participants’ understanding of program commitment, potential risks, benefits, and research activities. In order to participate, eligible families (a family triad: father, mother, and child) each needed to provide written consent to participate. Thus, in addition to parental consent, eligible children were asked to assent to their participation. The Institutional Review Board (IRB) at Texas A&M University reviewed and approved this research prior to beginning data collection. Additional IRBs at collaborating institutions also provided review and approval of research procedures. All research staff had completed the CITI (Collaborative Institutional Training Initiative) Program online training in ethics for social and behavioral research. 

### 2.6. Program Structure

The HEPP program was primarily a 6-week nutrition program with physical activity segments. Components included in-person group sessions, phone calls, and at-home activities, which supported the program’s overall theme. Weekly themes supported the overall theme. Table 1 presents the weekly themes. There was a different theme each week (e.g., Healthy Traditions for Healthy Families, Cooking with Papi is Fun) to ensure cohesion within and across group sessions. 

The overall theme was to embrace existing health-promoting traditions while encouraging new healthy traditions within families—a strategy guided by a strengths-based approach and the importance of feedback loops from the FAMILI approach [11] and prior research with Latino fathers [34,48]. Previously, a different nutrition program with multicultural children, including Latino children, focused on traditional foods or food practices to connect with program participants, reinforce traditional values, and support behavior changes related to nutrition [49]. In addition, findings from qualitative studies with Latino/a parents, including fathers, have documented the importance of incorporating traditional foods into programs and creating opportunities to continue food traditions with their children [48,50]. HEPP integrated traditional foods and food practices and traditional games as a starting point for promoting nutrition and physical activity behaviors with a strengths-based approach. 

### 2.7. Program Components

Overall structure—HEPP included in-person group sessions, weekly check-in phone calls, and at-home activities, which were scheduled between group sessions.

#### 2.7.1. Group sessions

The program consisted of six weekly group sessions. Table 2 shows an example of one group session for week 3. 

Group sessions primarily focused on nutrition, though each session included a physical activity break. Within each session, participants completed the following program activities in this order: food and beverage tastings (with three healthy recipes and a mini-nutrition educational lesson, Table 3), welcome, interactive lesson (participants gained knowledge and skills related to family functioning and nutrition, Table 4), physical activity break with active play (participants moved or exercised together), cooking lesson (participants prepared one healthy recipe with promotora, Table 3), eating together lesson (participants enjoyed a recipe they prepared with goal-setting, Table 5), and farewell with take-home messages (promotoras summarized sessions and provided reminders about at-home activities and the next session, Table 6). The raffles with prizes happened at the end of the group session. Table 3, Table 4, Table 5 and Table 6 present details on the cooking lessons, eating together lessons, respectively, and these lessons are discussed later in the manuscript. 

Some sessions were geared only for fathers and participating child (e.g., family dyad) and other sessions for the family triad (father, mother, and participating child). Half of the sessions were designed explicitly to support the father’s nutrition and physical activity. Decisions to focus sessions on the fathers (weeks 3, 5, and 6) or both parents, as coparents and partners (week 4), were made based on formative work and promotoras’ experience and their feedback. 

Table 4 outlines the interactive lessons, including the activity and key messages, by week. The first session offered kick-off festivities to celebrate the program and encouraged families to return for the next session; all activities were designed for the family triad of a father, child, and mother. The second session focused on the family as a system and engaged the family members to identify individual and shared family values. Identification of their values as strengths facilitated discussion about healthy traditions within families. In addition, this session included specific activities to directly engage children. Overall, the first two sessions were important for building trust and rapport initially and encouraging the mothers to support the fathers’ participation in designated father-child sessions. Again, decisions to structure group sessions were based on formative work and promotoras’ insights. The third session was the first session for the father-child dyad (Table 2). Week 3 recognized the special relationship between fathers and children and demonstrated their mutual influence on each other and their family. The fourth session was the culminating point of the program, where participants applied knowledge and practice skills from weeks 1 through 3. The fourth session engaged the coparents (fathers and mothers) separately from the children to support them as coparents and partners, which families identified as important in the formative work. The fifth session provided an opportunity to focus on nutrition education tailored to the fathers’ routines related to work or the weekends. The final session acted as a transition to review previous content, set up participants to sustain any behavioral changes post-program, and celebrate families’ journeys in the program. 

Promotoras delivered the group sessions using a leader’s guide, which contained the curriculum with scripts, activities, and worksheets. Participants were given copies of materials for activities in their preferred language and encouraged to keep materials in their Family Guide. Each family received two binders, a Family Guide for the participating child and another Family Guide for the parents and entire family. Families were asked to bring their Family Guide to all group sessions. 

During each group session, promotoras guided participants through experiential education, skills building, and goal-setting activities across the interactive, cooking, and eating together lessons (Table 3, Table 4 and Table 5). The amount of time for each component varied across activities. But, proportionately more time was dedicated to the cooking lesson. The cooking lesson facilitated families cooking together and supported the primary outcome of improved dietary intake of fruits and vegetables. As others have described, families cooking together is a way to promote collaboration, communication, and creativity among family members [51]. Each group session included a physical activity break (≈15 min). Throughout the lessons and during transitions, promotoras engaged with families one-on-one, tailoring information to individual parents or children within and across families [36]. Table 2 presents details for an entire group session for week 3. Participants also received small items that served as cues to action for initiating and sustaining nutrition and physical activity behaviors. For example, the program included branded water bottles and Fresh Baby (Petoskey, MI, 49770 USA) nutrition education materials like cutting mats, MyPlate (MiPlato) plates, drawstring backpacks. At the end of the group sessions, promotoras introduced the at-home activities for parents and children in the Family Guide, which always included self-monitoring worksheets, educational handouts, and instructions and supplies for new activities to promote co-participation in nutrition and physical activity. 

#### 2.7.2. Weekly Phone Calls

In addition to the weekly in-person group sessions, the promotoras completed weekly check-in phone calls with mothers. Each call included reviewing which recipes, nutrition, and physical activity-focused activities the families tried at home, how they modified the recipes or activities, or their reasons for not trying recipes or activities. (All recipes and activities were contained in the Family Guide.) Promotoras also asked participants to identify the most valuable aspect of the last group session, share any feedback or suggestions for improvement, and if they shared any of the information or activities with their social networks. 

#### 2.7.3. At-home Activities

The at-home component included additional activities for families to complete in between sessions. For example, as part of week 3, children received an apron and special fabric markers to customize aprons, which they could use during the subsequent cooking lessons. The “Homefun” component from the *Aventuras para Niños* Program provided inspiration for at-home activities related to nutrition and physical activity [36]. Handouts and worksheets were pre-printed and added to the Family Guide at each group session. Table 6 presents details on the at-home activities related to nutrition (Information on the at-home activities related to physical activity have been published separately [52]).

### 2.8. Program Duration, Dose, and Delivery

The literature informed decisions regarding duration, number, frequency, and length of sessions, and an appropriate amount of total dose delivered. The rationale was based on maximizing engagement and retention while not overburdening families with an extended program or exceeding available resources. The program lasted 6 weeks and was made up of 6 weekly group sessions (2.5 h per group session). The estimated dose delivered for fathers and children is 900 min (15 h); the estimated dose for mothers is 450 min (7.5 h). Because the program was father-focused and family-centered, there were more sessions for the fathers and children. For example, three sessions focused exclusively on fathers and children (sessions 3, 5, and 6). The estimated dose does not include additional program exposure via check-in phone calls or text messages, or time spent engaged in the at-home activities between group sessions. 

Table S2 provides detailed data on number, frequency, and length of contacts from previous nutrition and physical activity programs, and programs designed especially for Latino families, such as *Entre Familia: Reflejos de Salud* (14 weekly contacts @ 1.2 h/session, 16.5 h estimated dose delivered [42]), *Aventuras para Niños* (7 monthly contacts @ 1.5 h/session, 10.5 h estimated dose delivered [36]), *Padres Preparados, Jóvenes Saludables* (8 weekly contacts @ 2.5 h/session, 20 h estimated dose delivered [53]), *Abriendo Caminos* (6 weekly contacts @ 2 h/session, 12 h estimated dose delivered [54]), Community Outreach Obesity Prevention Trial (COOPT) (16 contacts @ 1.5 h/session, frequency of contacts not reported, 25 h estimated dose delivered) [43]), and other family-based nutrition programs, including Healthy Dads, Healthy Kids (HDHK) with white fathers and children in Australia (10.5 h estimated dose delivered) [55,56] and Growing Right Onto Wellness (GROW) with children from Latino or African American families in the U.S. (18 h estimated dose delivered [2]). In summary, prior family-based and family-centered programs planned for a dose delivered between 10.5 and 25 h (Table S2). Thus, HEPP’s estimated dose delivered of 15 h is like previous studies. Moreover, process evaluation results for *Entre Familia: Reflejos de Salud* showed that retention was about 88% [57], which further bolstered the decision to design a 6-week program, with 6 weekly contacts, and an estimated dose delivered of 15 h.

The team of promotoras delivered all group sessions in person. The research team delivered the program in July 2019 through May 2020. But the COVID-19 pandemic disrupted some program activities scheduled in mid-to-late March and through May. Changes were made to comply with policy changes at Texas A & M Univeristy. The promotoras scheduled weekly sessions consecutively on Saturdays at the Endowment Center in San Carlos, except for major holidays. The Endowment Center is a local community center in Hidalgo County, equipped with a large kitchen, flexible meeting rooms, and outdoor recreational areas. This location is convenient and considered a trusted and safe place by community members. Group sessions were offered on Saturday mornings (10:00 a.m.–12:30 p.m.) or afternoons (2:00 p.m.–5:30 p.m.). Previous research documented the importance of scheduling program sessions at times that facilitate engagement and active involvement of fathers and their families, such as times when fathers are not working [32]. Families described weekends as opportunities for family time and acceptable options for group sessions from the formative work. The promotoras encouraged families to attend the same session each week, but the promotoras allowed families to change sessions when needed to encourage retention. 

Consistent with participants’ preferences for communication, promotoras presented all activities verbally in Spanish with props and visual aids, including large posters and written materials for participating families. However, in one-on-one conversations, promotoras often spoke English, particularly with the children. Promotoras also provided parents and children with large binders for written program materials. This binder was referred to as the Family Guide. Parents and children chose which language they preferred for the written materials for their Family Guides. Generally, children requested English written materials, and parents asked for Spanish written materials. The parents and children added new written materials to their binders each week. Materials supported the in-person group sessions and at-home activities. 

### 2.9. Additional Considerations

Because HEPP was a family-centered program, accommodations were made to engage the mothers. The literature on father engagement and family-centered approaches also supports engaging the children’s mothers [12]. Mothers participated in the group sessions during weeks 1, 2, and 4. They did not participate in weeks 3, 5, and 6, because those sessions were father-focused sessions. Half of all sessions placed additional focus on fathers. During the father-focused sessions (weeks 3, 5, and 6), promotoras engaged the mothers separately in *Charlas* (or informal conversations), which are a culturally relevant way to engage Latino/a adults in educational activities. Charlas have been used as community-based health promotion and disease prevention programs with Latino/a adults and sometimes have been combined with a promotora model [58,59]. Each Charla was an educational session focused on a non-nutrition topic and included prizes for participation. The Charla activities were held on-site at the community center but separately from the group session. Importantly, during the father-focused sessions, fathers and children prepared a plate of food from the group session for the mothers. Children temporarily left the group session to present the plated food to their mothers. The mothers enjoyed their food together after the Charla, while fathers and children continued their group sessions in a different room. Mothers reunited with the rest of their families at the end of the group session. 

### 2.10. Nutrition Curriculum

The nutrition curriculum consisted of all materials and activities from the in-person group sessions and at-home materials and activities in the Family Guide. Two components of the group sessions focused explicitly on nutrition: cooking lessons and tasting recipe “mini” nutrition education lessons. Table 3 presents details on the activities and key messages related to nutrition, including the main recipes, which were used in the cooking lessons, and the three tasting recipes presented before the group session. 

However, there was an implicit focus on nutrition in the eating together and goal setting component of the program. In the eating together lessons, families had an opportunity to discuss food and nutrition while eating the recipe they prepared in the cooking lessons. Table 5 presents details on the eating together lessons with goal-setting activities. 

Nutrition principles also were incorporated throughout other parts of the group sessions aside from the cooking lessons. For example, nutrition education was part of the farewell lessons (e.g., summarizing key nutrition messages from the cooking lesson) and the focus of one interactive lesson for week 5 (Table 4). For example, the game ¡*Vamos a Jugar Pirinola*! (Let’s Play Pirinola!), which is a version of a traditional game *Toma Todo*, included game cards with nutrition facts for parents and children. In addition, the at-home activities, such as Papi’s Little Chef, support increases in nutrition knowledge, attitudes, and self-efficacy related to eating fruits and vegetables (Table 6). 

HEPP focused on nutrition principles based on MyPlate [60]: consume a variety of foods within and across food groups (fruits, vegetables, grains, protein foods, and dairy); consume fruits and vegetables in any form (e.g., fresh, canned, frozen) and in any preparation (e.g., cooked, raw); focus on whole fruits and vegetables with little added salt, sugar, or fat; emphasis on dark green, red, and orange vegetables, which tend to be under-consumed by U.S. individuals [61]; beans, peas, and lentils are unique foods and an excellent source of nutrition; make half of the grains whole grains; and consume a variety of protein-rich foods, including beans, nuts, seeds, and soy products, seafood, and lean meats. HEPP emphasized nutrition principles to support eating more fruits and vegetables. Prior research with Latino families reported a need to promote increased fiber intake [57], and given the suboptimal intakes of dietary fiber for the U.S. population generally, fiber-rich foods were intentionally prioritized in the nutrition curriculum. Notably, the nutrition curriculum balanced traditionally preferred foods (e.g., avocado, cabbage) and food practices (e.g., preparing agua frescas and licuados, blending avocado in a molcajete to make guacamole), which tend to be more valued by Latino/a individuals, and foods and food practices that are more familiar with U.S. culture in general, as others have done [49].

The team intentionally developed recipes to reflect the sociocultural preferences of the program participants, accommodate resource limitations within households and the community, and minimize requirements for specialty appliances in the group sessions or at home [53]. Prior research conducted by the authors has described strengths and limitations of the resources within the community food environment [62,63], household food environments [29,64,65], and the physical activity environments [20,66,67,68,69]. Most recipes were vegetarian or plant-based, and required no animal proteins, such as meat, fish, or eggs. The research team completed nutrition analysis of recipes and calculated estimated cost of recipes (by serving and total cost), based on retail prices of local grocery stores. Recipes incorporated ingredients or foods, which are part of the food packages in WIC (Special Supplemental Nutrition Assistance Program for Women, Infants, and Children) and commonly purchased on the Lone Star card (via Electronic Benefits Transfer in the Supplemental Nutrition Assistance Program in Texas). Recipe development was an iterative process of creation, review, analysis, tests, and revision. In addition, for all recipes, the instructions included substitutions for some ingredients to accommodate variations in access to food, household food availability, and utilization of food, and ideas for how the recipe could be used in a “twist” as part of different snacks or meals. 

The main recipes, which were used in the cooking lessons (Table 3), appealed to different individuals in the family. For example, certain recipes appealed more to the preferences of fathers (weeks 2 and 3 with tacos and tostadas), mothers (week 4 spinach salad), and children (week 5 vegetable pinwheels and week 6 tuna salad “boats”). In addition, the main recipes selected for the cooking lessons facilitated skills-building over time. The first recipe used in the cooking lesson included basic skills, such as washing, peeling, and chopping fresh vegetables. Recipes required additional skills over time, with the more complicated recipes, such as pinwheels and the tuna salad boats, which were placed toward the end in weeks 5 and 6. 

All cooking lessons featured a “spotlight” fruit or vegetable prominently in the main recipe (Table 3). The six spotlight fruits and vegetables were: garbanzo bean (chickpea), jicama, cabbage, spinach, sweet potato, and avocado. Spotlights intentionally focused on vegetables from “under-consumed” food groups such as dark green and orange vegetables [61], and health-promoting cruciferous vegetables, which contain beneficial nutrients including fiber. Jicama and avocado were included because they are nutritious, affordable, and socially and culturally preferred by families in South Texas colonias.

Additional recipes, called tasting recipes, were presented to families as they entered the community center or the program space (Table 3). The promotoras prepared tasting recipes prior to the start of the group session. There were three tasting recipes at each group session: (1) a child-focused snack with fruits or vegetables, (2) a beverage, which was either an agua fresca (naturally flavored fruit or vegetable beverage made with water and fresh juice) or licuado (a thick blended drink or “smoothie” made with whole fruits or vegetables and sometimes with milk), and (3) a vegetable-focused side dish, with options to become a main dish. At least two of the three tasting recipes featured the spotlight fruit or vegetable for the next week’s group session. In this way, the tasting recipes acted as a preview for the spotlight in the next group session. Families were provided with copies of all recipes for each session, including the main recipe in the cooking lesson and the three tasting recipes, in the Family Guide.

### 2.11. Physical Activity Curriculum

Although HEPP primarily focused on nutrition, a physical activity curriculum was developed as an integral part of the program. Detailed information on the development of the physical activity curriculum is available in a separate article [52]. Each group session included a physical activity lesson with active play lasting roughly 15 min. The physical activity lesson served as a break between the interactive lesson and cooking lesson. Like the nutritional components, physical activity lessons aimed to blend health-promoting traditional activities with family engagement or coparticipation between family members. In addition, week 4 had an extended physical activity lesson with the children, which lasted about 50 min. Importantly, allocating more time to physical activity for the children made time to focus on coparenting with the fathers and mothers during this lesson. The program also included at-home resources and additional activities in the Family Guide to promote physical activity at home.

### 2.12. Outcome and Process Evaluation

While the focus of this manuscript is not program evaluation, this section describes the primary and secondary outcomes and measurement of outcomes and a brief overview of the data collected for process evaluation. Table 7 presents the measures and timing for the evaluation.

The pre- and post-test measures were collected before and after the program. Data were collected at two separate time points (A and B) to collect dietary intake and physical activity data over a seven-day period. Data collection techniques included interviewer-administered surveys, more objective techniques like reflection spectroscopy with the Veggie Meter for fruits and vegetable intake, accelerometry for physical and sedentary activity, anthropometry for height and weight), and subjective techniques like semi-structured interviews (qualitative data) for the process evaluation. The nutrition survey included questions to assess psychosocial indicators related to nutrition, eating behaviors, and food practices, and there was a similar survey for physical activity. Semi-structured interviews were used to learn more about participants’ experiences in the program as part of the process evaluation. All measures were collected for all individuals: fathers, children, and mothers (Table 7).

HEPP targeted behavioral changes related to nutrition and physical activity among Mexican-heritage fathers and their children. The primary outcome was a change in dietary intake of fruits and vegetables for the fathers and children between the pre- and post-test (about six weeks), measured using reflection spectroscopy and the skin carotenoid score obtained from the Veggie Meter^®^ (Longevity Link, LLC., http://www.longevitylinkcorporation.com/contact.html, accessed on 26 July 2021, Salt Lake City, UT, USA ). Prior research has shown preliminary evidence for feasibility and initial validity of using the Veggie Meter with a racially/ethnically diverse group of participants [70]. Secondary outcomes included: (1) change in the minutes of light and moderate physical activity as measured with ActiGraph GT9X accelerometers, (2) change in overall family functioning, as measured by the Family Adaptability and Cohesion Scale (FACES IV), the English and Spanish versions [41,71], and (3) change in weight status, based on body mass index, and calculated with measured weight (in kilograms) and height (in meters). Additional data were collected on child demographics (age and gender), food insecurity (measured using a modification to the U.S. Household Food Security Survey Module [72]), and psychosocial indicators related to eating and activity behaviors (e.g., self-efficacy, knowledge, attitudes, etc.), eating and activity behaviors, and food practices.

For process evaluation, promotoras collected data on fidelity (the extent to which the group sessions were implemented as designed), dose delivered (delivery of group session components), and dose received (participant attendance at group sessions and participation throughout group session). More detail is provided next. 

Dose delivered was evaluated using an observational checklist for each session. Each checklist included a full list of session components. For each of the components, promotoras were responsible for reporting if the component was delivered or not delivered, and when delivered, the extent of modifications, if any, that were made to each of the components. When any modifications were made, promotoras were instructed to provide a detailed description of the modification and their rationale for modification. 

Data to evaluate dose received was collected in two ways. First, participants self-reported attendance at group sessions using printed sign-in sheets, which were completed at the start of group sessions. Second, participants completed a self-assessment of program delivery at the end of group sessions. The checklist included items to document who (father, mother, child) participated in each of the group session activities. In addition, the checklist included two questions: (1) “What questions, concerns, or suggestions, if any, do you have about today’s session”? (2) “What else would you like to share, if anything”? For the three father-child sessions, when the mothers participated in Charlas, mothers completed a different self-assessment for them to report on dose delivered.

Additional evaluation data were collected. For example, for every session, children completed a brief questionnaire to report on the food’s appearance, taste, willingness to eat that food at home or school if that food was available and provide their overall opinion of the food items. This self-assessment provided information on the general acceptability of the foods from the children’s perspectives.

Approximately three to four months after the program was completed, the promotoras contacted participants to complete maintenance measures, as a short-term follow up after the program (Table 7). At that time, the promotoras conducted surveys as part of outcome evaluation, and semi-structured, in-depth interviews to provide context for interpreting the evaluation data. All individuals (fathers, mothers, and children) participated in the maintenance data collection. 

The maintenance data collection occurred during two different home visits. During the first visit, promotoras completed a nutrition and physical activity surveys, Veggie Meter scans (to measure dietary intake of fruits and vegetables) and began physical activity accelerometer tracking with each of the participating family members (father, mother, and child), respectively. During the second and final home visit, promotoras ended the physical activity accelerometer tracking with each of the participating family members. Additionally, promotoras conducted separate in-depth interviews with each of the participating family members to capture qualitative data related to their: (1) reasons for enrollment or participation, (2) sustained behavioral changes related to nutrition and physical activity, and (3) changes in their role within the family, (4) shifts in family functioning post-program, and (5) what they shared with their social networks. Promotoras conducted the interviews using an interview guide and audio-recorded the interviews.

## 3. Discussion

In summary, this manuscript presented the rationale and design for Haz Espacio para Papi (HEPP, Make Room for Daddy!), a family-centered, father-focused behavioral program for Mexican-heritage families living in border colonias. Prior research has discussed important ways that Latino fathers can influence their families’ health and well-being, including related to nutrition and physical activity [48,73,74,75]. But Latino fathers have been largely overlooked in nutrition and childhood obesity-related research [22,34]. At the same time, for nearly ten years, there have been calls for father-focused programs and family-centered programs with fathers to promote nutrition within families [12,55,76,77]. But, with few exceptions, very few behavioral programs in nutrition have prioritized Latino fathers and their families; one exception is *Padres Preparados, Jóvenes Saludables*, led by Hurtado and colleagues [53]. Given the unique strengths and limitations within the border colonias, and in engaging specifically with Latino fathers, this manuscript offers important insights about behavioral programs for nutrition and physical activity.

First, there are many unknowns for how best to engage and support Latino fathers in behavioral programs in nutrition [22,34]. However, the literature provides evidence regarding the potential benefits of a family-centered, or as described by Ayala and colleagues, a “whole family” approach that engages the family as a system [35,37]. 

HEPP integrated a family-centered approach from start to finish. For example, the group sessions were held in a festive and family environment with refreshments, music, colorful decorations, and end-of-session raffles with prizes selected with fathers, mothers, and children in mind. Interactive activities used in lessons included a combination of active play and movement, games, both traditional (e.g., Lotería, Toma Todo) and games popular in the U.S. culture (e.g., Trivia style game), arts and crafts (or creative activities), which engaged all family members, including additional children who wanted to opt out of the on-site child care and stay with their parents and sibling during the group sessions. A separate manuscript discusses other strategies for supporting behavior change with Latino fathers and their families [22]. The family systems approach to behavioral program is supported by National Institute of Minority Health and Health Disparities (NIMHD)’s call for research to advance the science of health disparities and achieve health equity [23,24,25]. 

Second, within Latino families, mothers serve as gatekeepers in different ways. Prior research has discussed the moderating influence of Latino fathers on nutrition-related behaviors, which is related to authority and control. However, more attention has been paid to the negative rather than the positive aspects of their involvement [78]. Research projects have historically not engaged with fathers and have primarily relied on mothers to report for the entire household or family, which means that Latino fathers, in particular, have not had many opportunities to share their perspectives related to nutrition in their families [22]. Preliminary observations from our formative work indicated the importance of directly connecting with fathers, given their varied reasons for why they may demonstrate a lack of motivation and interest. One explanation may be that their partners (children’s mothers) have not provided social support and as a result, fathers have not felt comfortable engaging with their families around nutrition-related behaviors. Not much is known about Latino fathers’ motivation or interest because they have not been directly asked or their partners have been asked to speak for them. Additional research that directly engages with Latino fathers would provide important insights for how to customize behavioral programs for Latino fathers and their families. 

HEPP explicitly focused on the fathers, but in a way that valued them as coparents and partners [12]. This program intentionally addressed many challenges associated with the design, delivery and evaluation of childhood obesity prevention in family contexts [11,12]. Our novel approach represents what Panter-Brick and colleagues described as a “game-changer,” which they defined as “engaging unequivocally with coparents—rather than include just mothers and explicitly or implicitly marginalize fathers and other coparents, as in the bulk of parenting programs implemented to-date” [12] (p. 1205). While Ayala and colleagues have discussed “spousal interference” from the lens of Latina mothers [78], more research is needed to understand how gendered aspects of food parenting may marginalize or exclude fathers within Latino families [50,79,80,81,82], and on the other hand, unrealized opportunities to support positive food parenting (and physical activity parenting) within Latino families. In addition, future studies may explore how Latino fathers’ interest and motivation for behavior change are influenced by their partners.

Limitations included designing and implementing a program for one specific community in South Texas colonias and a subgroup of Latino families in that community. In addition, the program was designed for families with co-habitating parents (e.g., two-parent households). Future research is needed to design more universal programs that may be effective for varied family structures. While the HEPP approach, structure, and parts of the curriculum may be relevant for other border communities and Latino families, the HEPP program may not be translated to all border communities or all Latino families. Future research will need to consider unique strengths and limitations at the community, household, and family level in the adaptation process. Another limitation may be resource demands, including the costs of training (on nutrition, physical activity, motivational interviewing, program delivery, and logistics), program delivery itself, and the data collection associated with evaluation. 

Strengths of the HEPP approach to program design included the community-engaged research process, and collaboration with promotoras, and application of behavioral health theories and models, including FAMILI (Family-centered Action Model of Program Layout and Implementation) [11]. In the authors’ opinion, this approach facilitated development of a unique program that supported nutrition and physical activity within Latino families living in border colonias. Importantly, HEPP is first of a few behavioral nutrition programs that is father-focused and family-centered and designed specifically for Latino families. *Padres Preparados, Jóvenes Saludables* shared a similar approach, and their team completed a pilot study in the Spring of 2017 [53]. The HEPP program went beyond traditional nutrition education to prioritize building motivation and skills needed to enact behavior change. The design operationalized key theoretical constructs and created program activities to support changes in those constructs at the individual and family levels. HEPP is the only study of its kind designed with border communities.

Future research can use the HEPP approach and curricula as a starting point for designing context-specific and culturally-relevant programs for Latino families. Our extensive formative work, community-engaged, and theory-driven approach provide a roadmap for designing, implementing, and evaluating a family-centered program to support behavior change. This study fills a critical gap in understanding strategies for supporting a whole family approach for health promotion. In addition, the HEPP provides curricula for nutrition and physical activity, which can be valuable in supporting prevention of chronic diseases like obesity, diabetes, and other cardiometabolic conditions.

## 4. Conclusions

In closing, HEPP applied theories of community and father engagement and behavior change to develop a father-focused, family-centered program for Mexican-heritage families to support nutrition and physical activity. This kind of innovative program may be what’s required to “accommodate the ecologies of families and empower families in the process” [11] (p. 460). Future health promotion with Latino families can benefit by engaging parents and children together to achieve individual and collective goals related to nutrition and intentionally working with the mothers to support fathers as agents-of-change within their families.

## Figures and Tables

**Table 1:** Weekly themes by session.

Session	Theme
1	Welcome!
2	Healthy Traditions for Healthy Families
3	Cooking with Papi is Fun
4	Positive Parenting|Adventurous Children
5	Nutrition Basics
6	Healthy Families Going Forward

HEPP included six weekly sessions. Each session had a different theme. The weekly themes support the overall theme for the program, which is Embracing Existing Health-Promoting Traditions and Encouraging New Healthy Traditions with Families.

**Table 2:** Example of a week 3 “Cooking with Papi is Fun” with activities and estimated time.

Component	Session 3 Activity	Estimated Time
Tasting recipes lesson	Three recipes: Grilled cabbage with creamy chipotle dip (child-friendly snack) “Popeye” spinach smoothie (beverage) Grilled cabbage with salsa verde (vegetable-focused snack)	As families arrived
Welcome!	Greetings and Introduction	5 min
Interactive lesson	Superhero Drawing	20 min (5 min transition)
Physical activity break	Flip/Flop partner workout	13 min (2 min transition)
Cooking lesson	Main recipe: Chicken tostada with cabbage and mango-cucumber pico de gallo	55 min
Eating together	Icebreaker question: If you could have any superpower, what would it be? Why?	35 min
Goal setting	DIY (Do-It-Yourself) Invitation for family activity	5 min (5 min transition)
Farewell	Wrap-up and raffle	5 min

This table outlines the components of the group session for week 3 and includes estimated time for each component. The theme for this session was “Cooking with Papi is Fun”. For this week’s session, the spotlight fruit or vegetable was cabbage. Two of the tasting recipes and the main recipe (for the cooking lesson) featured cabbage prominently. The total time for the group session was 150 min.

**Table 3:** Spotlights, tasting recipes, main recipes, nutrition education and skills building for the cooking lessons.

Week	Spotlight	Tasting Recipes & Mini-Lesson	Main Recipe	Nutrition Education— Key Messages	Skills Building
1	Garbanzo bean	**Garbanzo bean** dip with carrots and jicama sticks Cucumber lime “icee” Tomato, **garbanzo bean**, and avocado salad Mini-Lesson: Introduce MyPlate basics, 5 nutritious food groups, and 5 key messages with each food group.	Red cabbage, carrot, and salad greens tossed salad with **garbanzo bean** and vinaigrette	1. Cooking together with variety of fruits and vegetables as family traditions (existing and new traditions). 2. Traditional foods and techniques can be part of healthy traditions. 3. MyPlate guidance: Benefits for eating more fruits and vegetables, and a variety of different types of vegetables in any form.	Learn how to: 1. Apply basic food safety/hygiene and food-handling practices. 2. Work with natural flavors, herbs, and spices. 3. Prepare and cut up vegetable safely. 4. Work with canned goods. 5. Make tossed salad. 6. Prepare a basic vinaigrette salad dressing. 7. Store vegetables for later and tips to minimize food waste.
2	Jicama	Roasted poblano dip with **jicama** and carrot sticks Basic yogurt smoothie **Jicama**, carrot, and orange salad Mini-Lesson: Identify energy, macronutrients, micronutrients in the Nutrition Facts Panels	Chicken tacos with **jicama**-cabbage slaw and homemade guacamole	1. Cooking together with a variety fruits and vegetables as family traditions (existing and new traditions). 2. Traditional foods (jicama, guacamole) and techniques (molcajete) can be part of healthy traditions. 3. Benefits of slaws. They are colorful, crunchy and tasty salads that are made with vegetables cut into matchsticks. 4. Slaws are nutritious. Reminder that MyPlate recommends half of the plate be fruits and vegetables and recommends for one to consume a variety of fruits and vegetables each day.	Reinforce previous skills Apply new skills. Learn how to: 1. Cut vegetables into matchsticks (thin strips). 2. Grind with traditional tool molcajete.
3	Cabbage	Grilled **cabbage** with creamy chipotle dip “Popeye” spinach smoothie Grilled **cabbage** with salsa verde Mini-Lesson: Review MyPlate focus on 5 food groups	Chicken tostada with **cabbage** and mango-cucumber pico de gallo	1. Cooking with children is a fun way to teach children traditions and create new traditions. 2. Children can help safely prepare fruits, vegetables, and canned goods and make nutritious foods. 3. Children eat more nutritious foods when they are more involved in the process. 4. Reminder that MyPlate offers recipe ideas.	Reinforce previous skills Apply new skills. Learn how to: 1. Prepare and cut fruits safely. 2. Store fruits for later and tips to minimize food waste.
4	Spinach	Garden cups with **spinach** ranch dip with broccoli and cauliflower Cucumber-mint-green grape agua fresca Wilted **spinach** with onion, tomato, and sweet potato (quelites-inspired) Mini-Lesson: Review Nutrition Facts Label on added sugar (limits, sources, and examples in ingredients).	**Spinach** salad avocado, jicama and mango with chicken and mango vinaigrette.	1. Cooking with children is a fun way to make healthy meals together. 2. Children taking the lead encourages the family to eat more nutritious foods. 3. Reminders about MyPlate recommendations for a healthy diet with balance, variety, and moderation.	Reinforce previous skills Apply new skills. Learn how to: 1. Prepare a more complex vinaigrette (mango) salad dressing.
5	Sweet potato	Mango and cucumber with chile and lime **Sweet potato** licuado (smoothie) **Sweet potato** bruschetta with avocado-cactus (nopalito) salad Mini-Lesson: Nutrients found in variety of colorful fruits and vegetables.	Vegetable pinwheels with **sweet potato**, black beans, and avocado.	1. Plant-based proteins (beans) with whole grains (tortilla) can provide essential amino acids the body needs (complementary proteins). 2. Vegetarian recipes can work with fresh, frozen, and canned or jarred vegetables. 3. Pinwheels can be simple and nutritious (with complementary proteins) snacks or lunches.	Reinforce previous skills Apply new skills. Learn how to: 1. Apply the pinwheel technique for child-friendly snacks and lunches
6	Avocado	**Avocado**-mozzarella-tomato skewers **Avocado**-banana licuado (smoothie) **Avocado**, black beans, and corn salad Mini-Lesson: Nutrition Facts Practice with Capri Sun.	Tuna salad “boats” (tuna salad with **avocado** and garbanzo beans served in cucumber boats)	1. Encouraging children to create edible sculptures helps promote healthier eating habits. 2. Protein-based salads are versatile options for on-the-go snack or meal. 3. A twist on tuna salad (addition of tuna to the garbanzo beans) allows more iron to be absorbed in the body. 4. Growing children need more iron.	Reinforce previous skills Apply new skills. Learn how to: 1. Create edible sculptures for child-friendly snacks and lunches.

This table presents the tasting recipes and main recipe by session, with nutrition educational messages and skills. The spotlight fruit or vegetable is shown in bolded text.

**Table 4:** Activities and key messages for the interactive lessons.

Session	Activity	Key Messages	Rationale	Inspiration
1	Do-It-Yourself (DIY) Decorating Family Guide	Celebrate uniqueness of each family Connect each family to program	Support families as a system	Aventuras para Niños Program [36]—Use of creative activities like the poster contests and redesigns of playgrounds
2	Values Exploration Card Sort	Identify unique values as strengths for family Connect values to traditions in families	Reflect on family strengths	Entre Familia: Reflejos de Salud Program [35]—Card-sort activity used in focus groups as part of program development
3	Superhero Drawing	Reinforce fathers distinct and important roles Model reciprocal relationship between fathers with children Show how fathers and children can be *catalistas* or change-makers for health in their families	Support father-child relationship	Aventuras para Niños Program [36]—Use of creative activities like the poster contests and redesigns of playgrounds
4	Rocks-in-a-Box Activity	Illustrate values and stressors in family life Demonstrate value of communication in coparenting	Support fathers as coparents	Activities with stones are commonly used in counseling and therapy (family psychology)
	Lotería (Bingo game) and Other Traditional Games with Active Movements	Provide active alternatives or changes to traditional cultural games Start conversations for parents to share active games (or continue traditions) with children	Support creativity, active play, and traditional games	Entre Familia: Reflejos de Salud Program [37]—Traditional game ( lotería) offered opportunity to engage children in traditional and new twists for active play
5	¡Vamos a Jugar Pirinola! Toma Todo (Take It All) Game	Show how small changes related to food make a big difference with snacks and food away from home	Develop new knowledge in nutrition	*Entre Familia: Reflejos de Salud* Program [37]—Traditional game (toma todo) offered opportunity for nutrition education
6	Jeopardy-style Trivia Game	Key messages from previous sessions	Review progress and identify strategies for sustaining behavioral changes after program	Familiar U.S.-style game offered opportunity to review key messages and transition from active to maintenance phase

All materials and activities in interactive lessons supported overall theme and achievement of outcomes related to nutrition and physical activity.

**Table 5:** Icebreaker questions and goal-setting activities for the eating together lessons.

Week	Icebreaker Question	Goal-Setting Activity	Purpose of Activity
1	If you could describe your day in one color, which color would choose and why?	Wish List (two wishes for self and family)	Identify and set goals related to program participation
2	If you could be any kind of animal, which animal would choose and why?	Using Rulers	Assess importance and confidence of behavior change using rulers and problem-solving to create a plan for making behavior change
3	If you could have any superpower, what would it be why?	DIY (Do-It-Yourself) Invitation	Create a family event to support nutrition within family
4	If you could have named yourself, what name would you have picked and why?	Setting Priorities 1.0	Set priorities with rules, stickers, and create a plan for action
5	If you could have dinner with any person, living or deceased which person would choose and why?	Setting Priorities 2.0	Set priorities with rules, stickers, and create a plan for action
6	How do you feel about your two wishes now that the program is complete?	Two Wishes 2.0 (Keep, Ditch, or Add)	Reflect on self and family’s journal and set new goals going forward to plan long term

The key message was the same for each eating together component: Eating together and talking as a family or embracing family meals as traditions.

**Table 6:** At-home nutrition activities for children and parents from the farewell lessons.

Week	Spotlight	Child-Focused Activity	Additional Activity
1	Garbanzo bean (chickpea)	I-Spy @ The Pulga (Flea Market)	Practice with Nutrition Facts Panel: V8 Juice
2	Jicama	Papi’s Little Chef	Practice with Nutrition Facts Panel: GoGurt
3	Cabbage	Grilling with Papi DIY (Do-It-Yourself) Decorating Aprons	Practice with Nutrition Facts Panel: Sunny D
4	Spinach	Scavenger Hunt @ A Grocery Store	Practice with Nutrition Facts Panel: Takis
5	Sweet potato	Breakfast with Papi	Practice with Nutrition Facts Panel: Rice Krispies
6	Avocado	Spotlight Snack	Practice with Nutrition Facts Panel: Coke

The farewell lessons included reminders about the at-home activities, which were included in the Family Guide. Each week included a new child-focused activity that was not part of the in-person group session (e.g., I-Spy), and an additional opportunity to practice reading and interpreting the Nutrition Facts Panel with a different food or beverage item (e.g., V8 Juice), which were commonly consumed by families in the study area. All recipes from the in-person group sessions, the main recipe and three tasting recipes, were also included in the Family Guide.

**Table 7:** Measures and timing for the evaluation.

Measure	Pre Test		Post Test		Short-Term Maintenance	
	A	B	A	B	A	B
Nutrition survey	X		X		X	
Family functioning survey		X		X		
Physical activity survey		X		X	X	
Demographic survey	X					
Food security survey	X					
Reflection spectroscopy	X	X	X	X	X	
Accelerometry	X	X	X	X	X	X
Anthropometry	X	X	X	X	X	
Semi-structured interview						X

This table presents data collection for the pre-test, post-test, and maintenance measures (3-4 months after program completion). An “X” indicates the timing of the different measures or assessments for the program. The Veggie Meter^®^ scans, powered by reflection spectroscopy, provided a biomarker for dietary intake of fruits and vegetables. The accelerometers provided an objective measure of physical activity behaviors. Anthropometry included measures of height and weight.

## Supplementary Materials

The following are available online at https://www.mdpi.com/article/10.3390/ijerph181910117/s1, Table S1: Selected examples of how program targeted theoretical constructs, Table S2: Characteristics of previous behavioral programs to estimate dose delivered.

## References

[1] Yackobovitch-Gavan M., Wolf Linhard D., Yackobovitch-Gavan M., Linhard D.W., Nagelberg N., Poraz I., Shalitin S., Phillip M., Meyerovitch J. (2018). Intervention for childhood obesity based on parents only or parents and child compared with follow-up alone. Pediatr. Obes..

[2] Po’e E.K., Heerman W.J., Mistry R.S., Barkin S.L. (2013). Growing right onto wellness (GROW): A family-centered, community-based obesity prevention randomized controlled trial for preschool child-parent pairs. Contemp. Clin. Trials.

[3] Stea T.H., Haugen T., Berntsen S., Guttormsen V., Øverby N.C., Haraldstad K., Meland E., Abildsnes E. (2016). Using the intervention mapping protocol to develop a family-based intervention for improving lifestyle habits among overweight and obese children: Study protocol for a quasi-experimental trial. BMC Public Health.

[4] Verbestel V., De Henauw S., Maes L., Haerens L., Mårild S., Eiben G., Lissner L., Moreno L.A., Frauca N.L., Barba G. (2011). Using the intervention mapping protocol to develop a community-based intervention for the prevention of childhood obesity in a multi-centre European project: The IDEFICS intervention. Int. J. Behav. Nutr. Phys. Act..

[5] Rhee K.E., Jelalian E., Boutelle K., Dickstein S., Seifer R., Wing R. (2016). Warm parenting associated with decreasing or stable child BMI during treatment. Child. Obes..

[6] Twiddy M., Wilson I., Bryant M., Rudolf M. (2012). Lessons learned from a family-focused weight management intervention for obese and overweight children. Public Health Nutr..

[7] Davison K.K., Gicevic S., Aftosmes-Tobio A., Ganter C., Simon C.L., Newlan S., Manganello J.A. (2016). Fathers’ representation in observational studies on parenting and childhood obesity: A systematic review and content analysis. Am. J. Public Health.

[8] Morgan P.J., Young M.D. (2017). The influence of fathers on children’s physical activity and dietary behaviors: Insights, recommendations and future directions. Curr. Obes. Rep..

[9] Morgan P.J., Young M.D., Lloyd A.B., Wang M.L., Eather N., Miller A., Murtagh E., Barnes A.T., Pagoto S.L. (2017). Involvement of fathers in pediatric obesity treatment and prevention trials: A systematic review. Pediatrics.

[10] Neshteruk C.D., Nezami B.T., Nino-Tapias G., Davison K.K., Ward D.S. (2017). The influence of fathers on children’s physical activity: A review of the literature from 2009 to 2015. Prev. Med..

[11] Davison K.K., Lawson H.A., Coatsworth J.D. (2012). The family-centered action model of intervention layout and implementation (FAMILI): The example of childhood obesity. Health Promot. Pract..

[12] Panter-Brick C., Burgess A., Eggerman M., McAllister F., Pruett K., Leckman J.F. (2014). Practitioner review: Engaging fathers—Recommendations for a game change in parenting interventions based on a systematic review of the global evidence. J. Child Psychol. Psychiatry.

[13] Keys E.M., Norris J.M., Cameron E.E., Bright K.S., Tomfohr-Madsen L.M., Benzies K.M. (2019). Recruitment and retention of fathers with young children in early childhood health intervention research: A systematic review and meta-analysis protocol. Syst. Rev..

[14] Davison K.K., Gavarkovs A., McBride B., Kotelchuck M., Levy R., Taveras E.M. (2019). Engaging fathers in early obesity prevention during the first 1,000 days: Policy, systems, and environmental change strategies. Obesity.

[15] Fabiano G.A., Caserta A. (2018). Future directions in father inclusion, engagement, retention, and positive outcomes in child and adolescent research. J. Clin. Child Adolesc. Psychol..

[16] Davison K., Kitos N., Aftosmes-Tobio A., Ash T., Agaronov A., Sepulveda M., Haines J. (2018). The forgotten parent: Fathers’ representation in family interventions to prevent childhood obesity. Prev. Med..

[17] Forster-Cox S., Torres E., Adams F. (2018). Essential roles of promotores de salud on the US-Mexico border: A US-Mexico border health commission perspective. Glob. J. Health Educ. Promot.—Spec. Issue.

[18] Nalty C.C., Sharkey J.R., Dean W.R. (2013). Children’s reporting of food insecurity in predominately food insecure households in Texas border colonias. Nutr. J..

[19] Sharkey J.R., Dean W.R., Nalty C.C. (2013). Child hunger and the protective effects of supplemental nutrition assistance program (SNAP) and alternative food sources among Mexican-origin families in Texas border colonias. BMC Pediatr..

[20] Umstattd Meyer M.R., Sharkey J.R., Patterson M.S., Dean W.R. (2013). Understanding contextual barriers, supports, and opportunities for physical activity among Mexican-origin children in Texas border colonias: A descriptive study. BMC Public Health.

[21] Sharkey J.R., Nalty C., Johnson C.M., Dean W.R. (2012). Children’s very low food security is associated with increased dietary intakes in energy, fat, and added sugar among Mexican-origin children (6–11 y) in Texas border colonias. BMC Pediatr..

[22] Johnson C.M., Sharkey J.R., Gómez L. (2021). Latino fathers as catalistas (agents of change): Strategies to support Latino fathers in childhood obesity prevention. J. Nutr. Educ. Behav..

[23] Alvidrez J., Castille D., Laude-Sharp M., Rosario A., Tabor D. (2019). The National Institute on Minority Health and Health Disparities research framework. Am. J. Public Health.

[24] Duran D.G., Pérez-Stable E.J. (2019). Novel approaches to advance minority health and health disparities research. Am. J. Public Health.

[25] Agurs-Collins T., Persky S., Paskett E.D., Barkin S.L., Meissner H.I., Nansel T., Arteaga S.S., Zhang X., Das R., Farhat T. (2019). Designing and assessing multilevel interventions to improve minority health and reduce health disparities. Am. J. Public Health.

[26] Johnson C.M., Sharkey J.R., Dean W.R., St. John J.A., Castillo M. (2013). Promotoras as research partners to engage health disparity communities. J. Acad. Nutr. Diet..

[27] Balls-Berry J.E., Acosta-Pérez E. (2017). The use of community engaged research principles to improve health: Community academic partnerships for research. P. R. Health Sci. J..

[28] Barkin S., Schlundt D., Smith P. (2013). Community-engaged research perspectives: Then and now. Acad. Pediatrics.

[29] Johnson C.M., Sharkey J.R., Dean W.R. (2011). It’s all about the children: A participant-driven photo-elicitation study of Mexican-origin mothers’ food choices. BMC Womens Health.

[30] St. John J.A., Johnson C.M., Sharkey J.R., Dean W.R., Arandia G. (2013). Empowerment of promotoras as promotora-researchers in the comidas saludables & gente sana en las colonias del sur de Tejas (healthy food and healthy people in south Texas colonias) program. J. Prim. Prev..

[31] Davison K.K., Charles J.N., Khandpur N., Nelson T.J. (2017). Fathers’ perceived reasons for their underrepresentation in child health research and strategies to increase their involvement. Matern. Child. Health J..

[32] Vollmer R.L., Adamsons K., Mobley A.R. (2019). Recruitment, engagement, and retention of fathers in nutrition education and obesity research. J. Nutr. Educ. Behav..

[33] Stahlschmidt M.J., Threlfall J., Seay K.D., Lewis E.M., Kohl P.L. (2013). Recruiting fathers to parenting programs: Advice from dads and fatherhood program providers. Child. Youth Serv Rev..

[34] O’Connor T., Perez O., Garcia I.C., Gallagher M. (2018). Engaging Latino fathers in children’s eating and other obesity-related behaviors: A review. Curr. Nutr. Rep..

[35] Ayala G.X., Ibarra L., Arredondo E., Horton L., Hernandez E., Parada H., Elder J.P., Elk R., Landrine H., American Cancer Society (2012). Promoting healthy eating by strengthening family relations: Design and implementation of the entre familia: Reflejos de salud intervention. Cancer Disparities: Causes and Evidence-Based Solutions.

[36] Duerksen S., Campbell N.R., Arredondo E.M., Ayala G.X., Baquero B., Elder J.P., Brettschneider W., Naul R. (2007). Aventuras para niños: Obesity prevention in the homes, schools, and neighborhoods of Mexican American children. Obesity in Europe: Young People’s Physical Activity and Sedentary Lifestyles.

[37] Ayala G.X., Ibarra L., Horton L., Arredondo E.M., Slymen D.J., Engelberg M., Rock C.L., Hernandez E., Parada H., Elder J.P. (2015). Evidence supporting a promotora-delivered entertainment education intervention for improving mothers’ dietary intake: The entre familia: Reflejos de salud study. J. Health Commun..

[38] Glanz K., Rimer B.K., Viswanath K. (2008). Health Behavior and Health Education: Theory, Research, and Practice.

[39] Davison K., Campbell K., Crawford D., Jeffery R. (2005). Opportunities to prevent obesity in children within families: An ecological approach. Obesity Prevention and Public Health.

[40] Kantor D., Lehr W. (1975). Inside the Family.

[41] Olson D. (2011). FACES IV and the circumplex model: Validation study. J. Marital. Fam. Ther..

[42] Horton L.A., Parada H., Slymen D.J., Arredondo E., Ibarra L., Ayala G.X. (2013). Targeting children’s dietary behaviors in a family intervention: “Entre familia: Reflejos de salud”. Salud Publica Mex..

[43] Yun L., Boles R.E., Haemer M.A., Knierim S., Dickinson L.M., Mancinas H., Hambidge S.J., Davidson A.J. (2015). A randomized, home-based, childhood obesity intervention delivered by patient navigators. BMC Public Health.

[44] Berge J.M., Wall M., Larson N., Loth K.A., Neumark-Sztainer D. (2013). Family functioning: Associations with weight status, eating behaviors, and physical activity in adolescents. J. Adolesc. Health.

[45] Vaughn A.E., Ward D.S., Fisher J.O., Faith M.S., Hughes S.O., Kremers S.P., Musher-Eizenman D.R., O’Connor T.M., Patrick H., Power T.G. (2016). Fundamental constructs in food parenting practices: A content map to guide future research. Nutr. Rev..

[46] Miller A., Franzen-Castle L., Aguirre T., Krehbiel M., Colby S., Kattelmann K., Olfert M.D., Mathews D., White A. (2016). Food-related behavior and intake of adult main meal preparers of 9–10 year-old children participating in iCook 4-H: A five-state childhood obesity prevention pilot study. Appetite.

[47] Heerman W.J., White R.O., Barkin S.L. (2015). Advancing informed consent for vulnerable populations. Pediatrics.

[48] Zhang Y., Hurtado G.A., Flores R., Alba-Meraz A., Reicks M. (2018). Latino fathers’ perspectives and parenting practices regarding eating, physical activity, and screen time behaviors of early adolescent children: Focus group findings. J. Acad. Nutr. Diet..

[49] Chen Q., Goto K., Wolff C., Bianco-Simeral S., Gruneisen K., Gray K. (2014). Cooking up diversity. Impact of a multicomponent, multicultural, experiential intervention on food and cooking behaviors among elementary-school students from low-income ethnically diverse families. Appetite.

[50] Lindsay A.C., Wallington S.F., Muñoz M.A., Greaney M.L. (2018). A qualitative study conducted in the USA exploring Latino fathers’ beliefs, attitudes and practices related to their young children’s eating, physical activity and sedentary behaviours. Public Health Nutr..

[51] Lukas C.V., Cunningham-Sabo L. (2011). Qualitative investigation of the cooking with kids program: Focus group interviews with fourth-grade students, teachers, and food educators. J. Nutr. Educ. Behav..

[52] Prochnow T., Umstattd Meyer M.R., Johnson C., Delgado H., Gómez L., Sharkey J. (2021). The development and pilot testing of the ¡Haz espacio para papi! program physical activity curriculum for Mexican-heritage fathers and children. Am. J. Health Educ..

[53] Zhang Y., Hurtado G.A., Reyes A.P., Brazys P.A., Perdue L., de Davila S.A., Florex R., Popeka J.M., Reicks M. (2019). Padres preparados, jóvenes saludables, a family-based program to prevent obesity among Latino early adolescents: Pilot test findings. J. Hum. Sci. Ext..

[54] Hannon B., Teran-Garcia M., Nickols-Richardson S.M., Musaad S.M., Villegas E.M., Hammons A., Wiley A., Fiese B.H. (2019). Implementation and evaluation of the abriendo caminos program: A randomized control trial intervention for Hispanic children and families. J. Nutr. Educ. Behav..

[55] Morgan P.J., Lubans D.R., Callister R., Okely A.D., Burrows T.L., Fletcher R., Collins C.E. (2011). The ‘Healthy dads, healthy kids’ randomized controlled trial: Efficacy of a healthy lifestyle program for overweight fathers and their children. Int. J. Obes..

[56] Morgan P.J., Lubans D.R., Callister R., Okely A.D., Burrows T.L., Fletcher R., Collins C.E. (2014). The ‘Healthy dads, healthy kids’ community randomized controlled trial: A community-based healthy lifestyle program for fathers and their children. Prev. Med..

[57] Schmied E., Parada H., Horton L., Ibarra L., Ayala G. (2015). A process evaluation of an efficacious family-based intervention to promote healthy eating: The entre familia: Reflejos de salud study. Health Educ. Behav..

[58] Balcazar H., Alvarado M., Alcalay R., Schindeldecker M., Newman E., Huerta E., Ortiz G. (2001). Salud para su corazón: Evaluating cardiovascular health outreach activities in the Latino community. Med. Am..

[59] Vincent D., McEwen M.M., Hepworth J.T., Stump C.S. (2014). The effects of a community-based, culturally tailored diabetes prevention intervention for high-risk adults of Mexican descent. Diabetes Educ..

[60] U.S. Department of Agriculture What’s on Your Plate?. https://www.myplate.gov/.

[61] U.S. Department of Agriculture, U.S. Department of Health and Human Services 2015–2020 Dietary Guidelines. https://health.gov/our-work/food-nutrition/previous-dietary-guidelines/2015..

[62] Sharkey J.R., Dean W.R., Johnson C.M. (2011). Association of household and community characteristics with adult and child food insecurity among Mexican-origin households in colonias along the Texas-Mexico border. Int. J. Equity Health.

[63] Sharkey J.R., Dean W.R., Johnson C.M. (2012). Use of vendedores (mobile food vendors), pulgas (flea markets), and vecinos o amigos (neighbors or friends) as alternative sources of food for purchase among Mexican-origin households in Texas border colonias. J. Acad. Nutr. Diet..

[64] Sharkey J.R., Dean W.R., St John J.A., Huber J.C. (2010). Using direct observations on multiple occasions to measure household food availability among low-income Mexicano residents in Texas colonias. BMC Public Health.

[65] Dean W.R., Sharkey J.R., Johnson C.M., John J.S. (2012). Cultural repertoires and food-related household technology within colonia households under conditions of material hardship. Int. J. Equity Health.

[66] Prochnow T., Umstattd Meyer M.R., Patterson M.S., Trost S.G., Gómez L., Sharkey J. (2021). Active play network influences on physical activity among children living in Texas colonias. Fam. Community Health.

[67] Umstattd Meyer M.R., Ylitalo K.R., Prochnow T., Gómez L.A., Sharkey J.R. (2020). Physical activity space methodology for assessment and prioritization (PASMAP): Combining systematic observations with community perceptions to identify community physical activity resource priorities. Health Place.

[68] Prochnow T., Umstattd Meyer M.R., Patterson M.S., McClendon M.E., Gómez L., Trost S.G., Sharkey J. (2020). Papás activos: Associations between physical activity, sedentary behavior and personal networks among fathers living in Texas colonias. Int. J. Environ. Res. Public Health.

[69] Prochnow T., Ylitalo K.R., Sharkey J., Umstattd Meyer M.R. (2019). Perceived physical activity barriers of Mexican-heritage sibling dyads. Am. J. Health Behav..

[70] Jilcott Pitts S.B., Jahns L., Wu Q., Moran N.E., Bell R.A., Truesdale K.P., Laska M.N. (2018). A non-invasive assessment of skin carotenoid status through reflection spectroscopy is a feasible, reliable and potentially valid measure of fruit and vegetable consumption in a diverse community sample. Public Health Nutr..

[71] Rivero N., Olson D.H., Martínez-Pampliega A. (2010). Spanish adaptation of the FACES IV questionnaire: Psychometric characteristics. Fam. J..

[72] U.S. Department of Agriculture Food Security—Survey Tools. Economic Research Service, U.S. Department of Agriculture. https://www.ers.usda.gov/topics/food-nutrition-assistance/food-security-in-the-us/survey-tools.aspx.

[73] Hofferth S.L. (2003). Race/ethnic differences in father involvement in two-parent families: Culture, context, or economy?. J. Fam. Issues.

[74] Villarruel F.A., Chahin J. (1997). Beyond the myths: Paternal values of Latino fathers. Mich. Fam. Rev..

[75] Parada H., Ayala G.X., Horton L.A., Ibarra L., Arredondo E.M. (2016). Latino fathers’ feeding-related parenting strategies on children’s eating. Ecol. Food Nutr..

[76] Freeman E., Fletcher R., Collins C., Morgan P., Burrows T., Callister R. (2012). Preventing and treating childhood obesity: Time to target fathers. Int. J. Obes..

[77] Khandpur N., Blaine R.E., Fisher J.O., Davison K.K. (2014). Fathers’ child feeding practices: A review of the evidence. Appetite.

[78] Schmied E.A., Parada H., Horton L.A., Madanat H., Ayala G.X. (2014). Family support is associated with behavioral strategies for healthy eating among Latinas. Health Educ. Behav..

[79] Tschann J.M., Martinez S.M., Penilla C., Gregorich S.E., Pasch L.A., De Groat C.L., Flores E., Deardorff J., Greenspan L.C., Butte N.F. (2015). Parental feeding practices and child weight status in Mexican American families: A longitudinal analysis. Int. J. Behav. Nutr. Phys. Act..

[80] Penilla C., Tschann J.M., Deardorff J., Flores E., Pasch L.A., Butte N.F., Gregorich S.E., Greenspan L.C., Martinez S.M., Ozer E. (2017). Fathers’ feeding practices and children’s weight status in Mexican American families. Appetite.

[81] Watterworth J.C., Hutchinson J.M., Buchholz A.C., Darlington G., Simpson J.A.R., Ma D.W.L., Haines J., Guelph Family Health Study (2017). Food parenting practices and their association with child nutrition risk status: Comparing mothers and fathers. Appl Physiol. Nutr. Metab..

[82] Davison K.K., Haines J., Garcia E.A., Douglas S., McBride B. (2020). Fathers’ food parenting: A scoping review of the literature from 1990 to 2019. Pediatr. Obes..

