# Supplementary material for: Designing for Multilevel Behavior Change: A Father-Focused Nutrition and Physical Activity Program for Mexican-Heritage Families in South Texas Border Communities
Source: Int J Environ Res Public Health. 2021 Sep 26;18(19):10117. doi: 10.3390/ijerph181910117 (PMC8508574; doi:10.3390/ijerph181910117)
Supplement: Supplementary file 1 [file ijerph-18-10117-s001.zip › ijerph-1337362-supplementary.pdf]

Supplementary Table S1. Selected examples of how program targeted theoretical constructs.

Theory	Theoretical construct	Definition of construct	Program component or activity
Social Cognitive Theory [38]	Self-efficacy	A person's confidence in being able to successfully perform a behavior	Promotoras created opportunities to practice new skills (e.g., hands-on activities) in interactive lessons, physical activity breaks, cooking lessons, and eating together lessons; Sequencing of skills (e.g., cooking lessons started with basic skills and increased in difficulty over time); Repetition of skills in a twist or variation (e.g., preparing a basic vinaigrette in session 1 and mango vinaigrette in session 4).
	Knowledge		Mini-educational tasting lessons to support increases in nutrition knowledge; Large visual aids (giant posters) with educational messages related to nutrition and physical activity; Small cues to action like MyPlate branded "goodies" with nutrition educational messages (e.g., Fresh Baby cutting mats, etc.); Focused session (week 5) to develop culturally-relevant nutrition knowledge in a fun and traditional game; Educational materials related to nutrition and physical activity contained in the Family Guide.
	Attitudes		Interactive lessons with focus on family's unique values and building culturally-relevant nutrition knowledge to shift beliefs related to nutrition and physical activity.
Circumplex Model [41]	Family functioning	Family cohesion: "emotional bonding that family members have toward one another."<br>Family flexibility: "the quality and expression of leadership and organization, role relationship, and relationship rules and negotiations."<br>Family communication: "the positive communication skills utilized in the couple or family system." (p. 65)	Special family dyad (father-child) or family triad (father-child-mother) activities in interactive lessons, physical activity breaks, cooking lessons, and eating together lessons to promote family cohesion; Promotoras' modeling open questions, curious tone, and non-judgmental responses to demonstrate positive communication in interpersonal interactions; Prompts to facilitate more positive family conversations during all lessons, especially in the eating together lessons with goal-setting; Eating together lessons included goal-setting and problem-solving with the support of promotoras to develop adaptability.

This table presents examples of how the program operationalized theoretical constructs and targeted theoretical constructs through program components or activities.

Supplementary Table S2. Characteristics of previous behavioral programs to estimate dose delivered.

Program	Focused on Fathers	Focused on Latino Families	Duration	Number of Contacts	Frequency of Contacts	Duration of Contacts	Estimated Dose Delivered
¡Haz Espacio para Papi! (HEPP)	Yes	Yes	6 weeks	6	Weekly	2.5 hours	15 hours*
Aventuras para Niños [36]	No	Yes	7 months	7	Monthly	1.5 hours	10.5 hours**
Healthy Dads, Healthy Kids (HDHK) [55,56]	Yes	No	7 weeks	7	Weekly	1.5 hours	10.5 hours
Abriendo Caminos [54]	No	Yes	6 weeks	6	Weekly	2 hours	12 hours
Entre Familia: Reflejos de Salud [42,57]	No	Yes	16 weeks	14	Weekly (tapered)	1.2 hours	16.5 hours
Growing Right Onto Wellness (GROW) [2]	No	No***	12 weeks	12	Weekly	1.5 hours	18 hours
Padres Preparados, Jóvenes Saludables [53]	Yes	Yes	8 weeks	8	Weekly	2.5 hours	20 hours
Community Outreach Obesity Prevention Trial (COOPT) [43]	No	Yes	6 months	16	NR	1.5 hours	25 hours

This table presents data for the estimation of dose delivered based on the number of contacts, frequency of contacts, and duration of contacts. Authors calculated estimated total dose using published data for behavioral programs and when needed Clinical Trials.gov (https://clinicaltrials.gov/). For programs with active and maintenance phases, the number of contacts were based on the active program phase only and did not consider the maintenance phase. Previous behavioral programs arranged from the least to greatest amount of estimated dose delivered.
*Estimated dose delivered for the fathers and children. The estimated dose delivered for the mothers 7.5 hours.
**Estimated dose delivered based on the micro program and not the macro programs.
***Program focused on Latino and African American children.

NR: Not reported
